# Prediction and Analysis of Retinoblastoma Related Genes through Gene Ontology and KEGG

**DOI:** 10.1155/2013/304029

**Published:** 2013-08-13

**Authors:** Zhen Li, Bi-Qing Li, Min Jiang, Lei Chen, Jian Zhang, Lin Liu, Tao Huang

**Affiliations:** ^1^Department of Ophthalmology, Ren Ji Hospital, School of Medicine, Shanghai Jiao Tong University, Shanghai 200127, China; ^2^Key Laboratory of Systems Biology, Shanghai Institutes for Biological Sciences, Chinese Academy of Sciences, Shanghai 200031, China; ^3^State Key Laboratory of Medical Genomics, Institute of Health Sciences, Shanghai Jiaotong University School of Medicine and Shanghai Institutes for Biological Sciences, Chinese Academy of Sciences, Shanghai 200031, China; ^4^College of Information Engineering, Shanghai Maritime University, Shanghai 201306, China; ^5^Department of Ophthalmology, Shanghai First People's Hospital, Shanghai Jiaotong University, Shanghai 200080, China; ^6^Department of Genetics and Genomic Sciences, Mount Sinai School of Medicine, New York City, NY 10029, USA

## Abstract

One of the most important and challenging problems in biomedicine is how to predict the cancer related genes. Retinoblastoma (RB) is the most common primary intraocular malignancy usually occurring in childhood. Early detection of RB could reduce the morbidity and promote the probability of disease-free survival. Therefore, it is of great importance to identify RB genes. In this study, we developed a computational method to predict RB related genes based on Dagging, with the maximum relevance minimum redundancy (mRMR) method followed by incremental feature selection (IFS). 119 RB genes were compiled from two previous RB related studies, while 5,500 non-RB genes were randomly selected from Ensemble genes. Ten datasets were constructed based on all these RB and non-RB genes. Each gene was encoded with a 13,126-dimensional vector including 12,887 Gene Ontology enrichment scores and 239 KEGG enrichment scores. Finally, an optimal feature set including 1061 GO terms and 8 KEGG pathways was obtained. Analysis showed that these features were closely related to RB. It is anticipated that the method can be applied to predict the other cancer related genes as well.

## 1. Introduction

Retinoblastoma (Rb) is a rapidly developing cancer in infants that develops in the cells of retina, the light-detecting tissue of the eye [1], which can be heritable or nonheritable. The most common and obvious sign of retinoblastoma is an abnormal appearance of the pupil, leukocoria, also known as amaurotic cat's eye reflex [2]. Retinoblastoma is rare and affects approximately 1 in 15,000 live births, but it is the most common inherited childhood malignancy. In China, around 1100 new cases are diagnosed each year, just second to that of India. Patients without diagnosis and being treated untimely would undergo enucleation or even die. In about two-thirds of cases, only one eye is affected (unilateral retinoblastoma); in the other third, tumours develop in both eyes (bilateral retinoblastoma). The number and size of tumours on each eye may vary [2].

As a kind of neural ectoderm tumor, heritable Rb is mainly caused by the mutation of Rb gene and dysfunction of tumor suppressor genes [3]. In these years, a rise in the number of cases was found, which was partly blue to the environmental pollution. The defective RB1 gene can be inherited from either parents; in some children, however, the mutation occurs in the early stages of fetal development [4]. Somatic amplification of the MYCN oncogene is responsible for some cases of non-hereditary, early onset, aggressive, and unilateral Rb. Although MYCN amplification accounted for only 1.4% of Rb cases, researchers identified it in 18% of infants diagnosed at less than 6 months of age. Median age at diagnosis for MYCN Rb was 4.5 months, compared with 24 months for those who had nonfamilial unilateral disease with two RB1 gene mutations [5]. Bilaterally affected individuals and 13%–15% of unilaterally affected individuals are expected to show an RB1 mutation in blood [6, 7]; the rest 85% of unilaterally affected patients were found not to carry either of their eye tumor RB1 mutations in blood; neither molecular testing nor clinical surveillance of siblings is required [8]. So to find more molecular markers or more effective prediction method is crucial for Rb diagnosis. 

System biology approaches for discovering cancer related genes have been reported [9–11]. The Gene Ontology (GO) is a major bioinformatics tool to unify the representation of gene and gene product attributes across all species [12]. GO terms have been used previously to characterize protein function and to elucidate trends in protein datasets [13]. In addition, it has been shown that GO annotations are good predictors of cancer genes [14]. The Kyoto Encyclopedia of Genes and Genomes (KEGG) pathway database is a widely used comprehensive inference for pathway mapping of genes.

Here, we developed a new systems biological measure to effectively and deficiently identify RB genes and their pathways. First, we identified 119 RB genes from the overlap of two gene expression studies of retinoblastoma. In order to identify GO terms and KEGG pathways that are distinct between RB and non-RB genes, 5,500 non-RB genes were randomly selected from the Ensembl genes. Then all the genes were encoded with 12,887 Gene Ontology enrichment scores and 239 KEGG enrichment scores. mRMR and IFS was used to rank these features. Dagging was employed as the prediction engine. Finally, 1061 GO terms and 8 KEGG pathways were obtained as the optimal features to discriminate an RB and non-RB gene, which has been shown to be closely related to RB.

## 2. Materials and Methods

### 2.1. Dataset

The 119 consistently differentially expressed genes between retinoblastoma and normal retina were obtained from the overlap between differentially expressed genes discussed in two gene expression studies of retinoblastoma [15, 16] (see Supplementary Material available at http://dx.doi.org/10.1155/2013/304029). In Chakraborty et al.'s study [15], there was a total of 10 RB samples and three normal retina samples. Human 19K cDNA microarray which was interrogating 19,000 human genes was used to get the expression profiling and the raw data were normalized by grid-wise normalization. In Ganguly and Shields's study [16], they investigated the gene expression of six matched RB tissues and normal retinal tissues with GeneChip Human U133 V2.0 microarray. Both the Affymetrix standard protocols and the standard model-based methods of robust multichip average were used. The values are background adjusted, normalized, and log transformed. There were 110 proteins corresponding to these 119 RB genes, which were regarded as positive samples in this study. The gene symbols were mapped to Ensembl proteins ID with the tool BioMart [17]. We randomly selected 110 × 50 = 5,500 non-RB genes from Ensembl as the negative samples. We refer the reader to [18] to deal with imbalanced data; all the negative samples were randomly split into 10 parts to comprise 10 datasets with the 110 positive samples. All the RB related genes and non-RB related genes are given in Supplementary S1.

### 2.2. Gene Ontology and KEGG Enrichment Scores

The Gene ontology enrichment score of a protein is defined as the −log10 of the hypergeometric test *P* value [19–21] of its direct neighbors in STRING network [22]. The higher the enrichment score of a certain Gene Ontology term, the more overrepresented it is. There were 12,887 Gene Ontology enrichment score features. In the same way, the KEGG enrichment score of a protein is defined as the −log10 of the hypergeometric test *P* value [19, 20] of its direct neighbors in STRING network [22]. The higher the enrichment score of one pathway, the more overrepresented the pathway is. There were 239 KEGG enrichment score features.

### 2.3. Feature Reduction

We calculated the Cramer's V coefficient [23, 24] between features and target variables. Cramer's V coefficient is a statistical measurement derived from the Pearson Chi-square test [25]. It ranges from 0 to 1. The smaller Cramer's V coefficient indicates weaker association. The features with Cramer's V coefficient small than 0.1 were removed. 

### 2.4. mRMR Method and Dagging

We used the minimum redundancy maximal relevance (mRMR) method to rank the importance of the features [26]. The mRMR method ranks features based on both their relevance to the target and the redundancy between features. A smaller index of a feature denotes that it has a better tradeoff between maximum relevance to the target and minimum redundancy. For detail, please refer to our previous works [21, 27–31]. 

Dagging is a metaclassifier that employs majority vote to combine multiple models derived from a single learning algorithm using disjoint samples [32]. For a training dataset *ℑ* = {*s*_1_, *s*_2_,…, *s*_*n*_}, *k* disjoint subsets of size *n*′ are constructed by randomly taking samples in *ℑ* without replacement, where *kn*′ ≤ *n*. Use a basic classifier to derive *k* classification models *M*_1_, *M*_2_,…, *M*_*k*_ from the constructed *k* disjoint subsets of *ℑ*. For a query sample, each of these models provides an output. The final predicted result is the class with most votes. In Weka 3.6.4 [33], the classifier “Dagging” implements the dagging classifier described above. In this study, it was employed as the classification model. For convenience, it was run with its default parameters. In detail, SMO is used as a basic classifier, and *k* is set to 10. In recent years, Dagging has been employed to deal with some biological problems [34–37]. Its performances in these studies show that it can be superior to some classic classifiers in some cases.

### 2.5. Ten-Fold Cross Validation and Incremental Feature Selection (IFS)

Ten-fold cross validation was often used to evaluate the performance of a classifier [38]. To evaluate the performance of the predictor, the prediction accuracy, specificity, sensitivity, and MCC (Matthews's correlation coefficient) were calculated as follows:

(1) $$\begin{array}{c} \text{accuracy}=\frac{\text{TP}+\text{TN}}{\text{TP}+\text{TN}+\text{FP}+\text{FN}},\\ \text{sensitivity}=\frac{\text{TP}}{\text{TP}+\text{FN}},\\ \text{specificity}=\frac{\text{TN}}{\text{TN}+\text{FP}},\\ \text{MCC}=\frac{\text{TP}\times \text{TN}-\text{FP}\times \text{FN}}{\sqrt{\left(\text{TP}+\text{FP}\right)\left(\text{TP}+\text{FN}\right)\left(\text{TN}+\text{FP}\right)\left(\text{TN}+\text{FN}\right)}}, \end{array}$$

where TP denotes true positive. TN denotes true negative. FP denotes false positive and FN denotes false negative.

Based on the features ranked by mRMR, we used incremental feature selection (IFS) [21, 28, 39, 40] to determine the optimal number of features. During IFS procedure, features in the ranked feature set are added one by one from higher to lower rank. A new feature set is composed when one feature is added. For each of the feature sets, a Dagging classifier is constructed and tested using ten-fold cross-validation test. Thus, an IFS table is obtained with one column being the index of the feature set and the other columns being the prediction accuracies, sensitivities, specificities, and MCCs. We then can get the optimal feature set, using the predictor that achieves the best prediction performance.

## 3. Results and Discussion

### 3.1. The mRMR Result

After running the mRMR software, we obtained two tables for each of the ten datasets (see Supplementary S2): one is called MaxRel feature table that ranks the features according to their relevance to the class of samples and the other is called mRMR feature table that lists the ranked features by the maximum relevance and minimum redundancy to the class of samples. In the mRMR feature table, a feature with a smaller index implies that it is more important for discriminating RB and non-RB genes. Such list of ranked feature was to be used in the following IFS procedure for the optimal feature set selection. 

### 3.2. IFS Result

**Figure 1:**
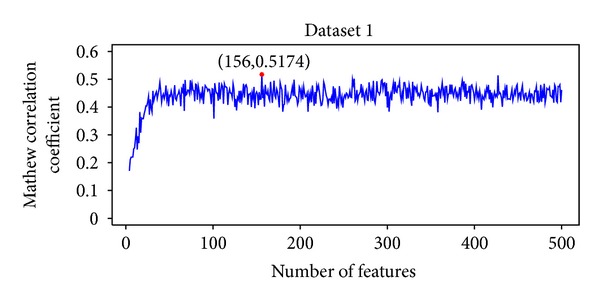
IFS curve for the first datasets. The maximal MCC was 0.5174 when 156 features were used.

By adding the ranked features one by one, we built 500 individual predictors based on 500 subfeature sets to predict RB genes for each of the ten datasets. We then tested the prediction performance for each of the 500 predictors and obtained the IFS results (see Supplementary S3). The IFS curves plotted based on the data of Supplementary S3 are shown in Figure 1. The IFS curve of dataset 1 is shown in Figure 1, we can see that the maximal MCC was 0.5174 when 156 features as given in Supplementary S3 were used. Such 156 features were regarded as the optimal feature set for dataset 1. Based on these 156 features, the prediction sensitivity, specificity, and accuracy were 0.5727, 0.9291, and 0.8697, respectively (Table 1). For the other nine datasets, the IFS results can be found in Supplementary S3 and corresponding IFS curves can be found in Supplementary S4. Finally, we took the union of optimal features for all the ten datasets as the final optimal feature set, which included 1061 GO terms and 8 KEGG pathways (see Supplementary S5). Hereafter, the further analysis was based on this final optimal feature set.

### 3.3. 119 RB Genes Enrichment Analysis

To compare the enrichment result of only positive sample and the selected GO and KEGG terms, we conducted the enrichment analysis for the 119 RB genes. The results showed that 12 GO terms were enriched significantly (Benjamini adjusted *P* value < 0.05; see Supplementary S6). Among them, two GO terms (GO:0007049: cell cycle and GO:0000087: M phase of mitotic cell cycle) were in our optimal feature set. For KEGG pathways, only hsa04110 (cell cycle) was significantly enriched (see Supplementary S6) and it has been included in our optimal feature set for distinguishing RB genes and non-RB genes, which suggested that these three enriched terms including GO:0007049: cell cycle, GO:0000087: M phase of mitotic cell cycle, and hsa04110: cell cycle are critical discriminators for RB genes and non-RB genes.

### 3.4. Analysis of the Optimal Feature Set

#### 3.4.1. GO Number and Percentage

To illustrate the biological meanings of the selected optimal feature subset, we firstly tried to classify GO terms in the optimal set into the three kinds: the biological process, cellular component, and molecular function GO terms. And the GO terms of the feature obtained by mRMR method were mapped to the children of the three root GO terms. The figures show the frequency of each GO term in the feature subset and display the ratio of the number of each GO term to the scale of the number of its children terms.

**Figure 2:**
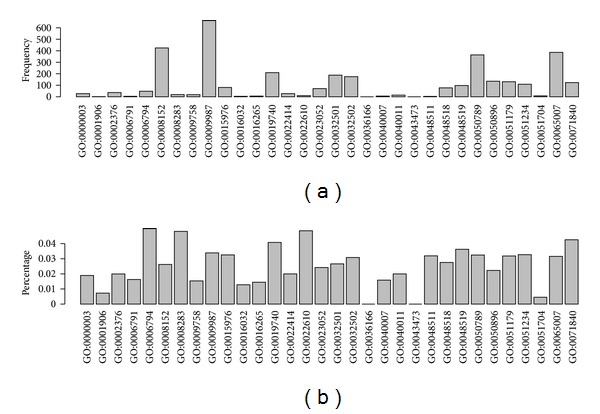
Illustrating the distribution of GO terms of biological process in the optimal feature set. (a) The frequency of GO terms of biological process. (b) The percentage of GO terms of biological process.

*(1)  Biological Process GO Terms*. From Figure 2, it can be seen that in the frequency of BP terms, the top five GO biological process terms are GO:0009987: cellular process (662), GO:0008152: metabolic process (425), GO:0065007: biological regulation (386), GO:0050789: regulation of biological process (364), and GO:0019740: nitrogen utilization (210). The inclusion of cellular process (GO:0009987), biological regulation (GO:0065007), and regulation of biological process (GO:0050789) within the top five frequencies of GO terms may suggest that these biological functions performed by certain proteins at the cellular level are very important in normal persons and may be dysfunctional in Rb patients.

For the percentage of BP terms, the top five GO biological processes are GO:0006794: phosphorus utilization (4.99%), GO:0022610: biological adhesion (4.85%), GO:0008283: cell proliferation (4.81%), GO:0071840: cellular component organization or biogenesis (4.26%), and GO:0019740: nitrogen utilization (4.08%). Phosphorus utilization provides cells phosphorylation sources and ensures regular cellular activities. From the GO biological process term percentage distribution, it can be seen that GO terms related with cell proliferation and biological adhesion are also highlighted, although their term numbers are less than those of the others. This indicates that proteins assigned with these two GO terms have relatively high influence on RB. For example, RB1 is a key regulator of cell proliferation and fate in retinoblastoma, phosphorylation of which can lead to conformational alterations and inactivates the capability of RB1 to bind partner proteins [41]. Cell adhesion also contributes to normal cells' exchange and communication. Epithelial cell adhesion molecule (EpCAM) can regulate expression of the oncogenic miR17-92 cluster in RB and thereby controls Rb cell proliferation and invasion [42].

**Figure 3:**
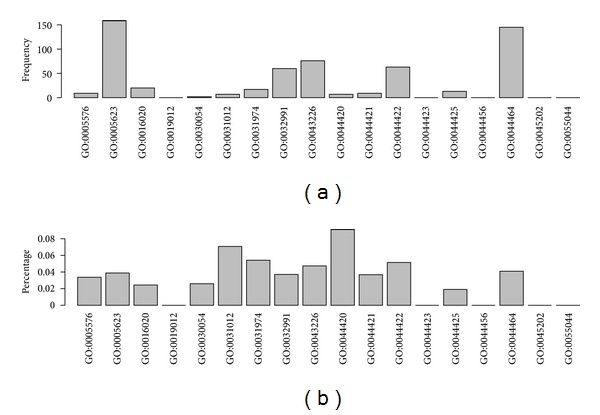
Illustrating the distribution of GO terms of cellular component in the optimal feature set. (a) The frequency of GO terms of cellular component. (b) The percentage of GO terms of cellular component.

*(2)  Cellular Component GO Terms*. In Figure 3(a), for frequency of CC terms, the top five GO cellular component terms are GO:0005623: cell (158), GO:0044464: cell part (145), GO:0043226: organelle (76), GO:0044422: organelle part (63), and GO:003299: macromolecular complex (60), mostly because of their large base numbers. In the percentage of CC terms, the top five GO cellular component terms also include GO:0044420: extracellular matrix part (9.09%), GO:0031012: extracellular matrix (7.07%), GO:0031974: membrane-enclosed lumen (5.41%), GO:0044422: organelle part (5.15%), and GO:0043226: organelle (4.73%).

Extracellular matrix is associated with cell adhesion mentioned in the last section. Inadhesive cells having destroyed extracellular matrix and no natural protections tend to be tumor cells under outside pressures. Here, from the percentage distribution, it is suggested that extracellular matrix was highly related with RB. Additionally, the inclusion of membrane-enclosed lumen, organelle, and organelle part indicated that cell organelles (with or without membrane) may involve in Rb too.

**Figure 4:**
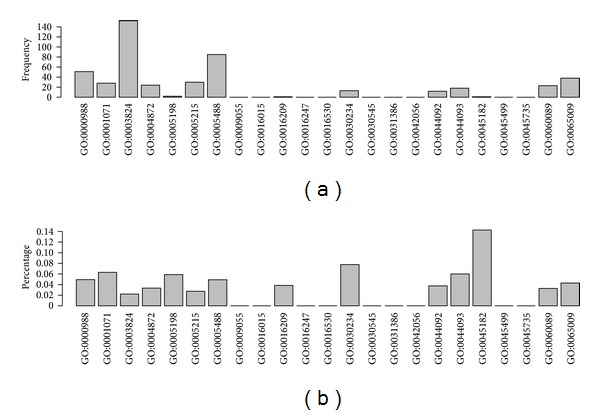
Illustrating the distribution of GO terms of molecular function in the optimal feature set. (a) The frequency of GO terms of molecular function. (b) The percentage of GO terms of molecular function.

*(3)  Molecular Function GO Terms*. In Figure 4, the top five GO molecular function terms of frequency are GO:0003824: catalytic activity (152), GO:0005488: binding (85), GO:0000988: protein binding transcription factor activity (51), GO:0065009: regulation of molecular function (38), and GO:0005215: transporter activity (30). Because of large base numbers, protein GO terms related to RB are relatively more enriched in the top five molecular function GO terms, especially in catalytic activity (GO:0003824) and binding (GO:0005488). Proteins assigned to these GO terms required interaction to carry out their structural or functional activities. This suggests that dysfunction of proteins assigned to these GO terms contributed profoundly to Rb tumorogenesis. The highlight of catalytic activity (GO:0003824) may be attributed to the fact that many Rb related proteins are involved in catalytic activities such as enzymes. The highlight of binding (GO:0005488) may be ascribed to the fact that proteins expressing specific function should regulate or interact with others through binding each other. Biological progresses such as phosphorylation and acetylation are critical in disease and both of them need certain enzyme to catalyze; for example, phosphorylated p53 can intiatie cell cycle arrest of abnormal cells and acetylated ones can cause apoptosis of injured cells [43, 44], and all these processes need binding and catalysis to execute function.

In Figure 4(b), the top five GO molecular function terms are GO:0045182: translation regulator activity (14.3%), GO:0030234: enzyme regulator activity (7.78%), GO:0001071: nucleic acid binding transcription factor activity (6.31%), GO:0044093: positive regulation of molecular function (6%), and GO:0005198: structural molecule activity (5.88%). Because of the large base number of the top five GO terms in frequency, they have relatively lower enrichment than the top five GO terms in percentage. But, the top five GO terms in MF percentage are all interrelated with these in BP percentage and CC percentage. For example, ribosome as a kind of organelle serves as translation vehicle in cells, which may somehow take part in the translation regulation. RB protein phosphorylation also needs enzyme to catalyze [45]. 

#### 3.4.2. The KEGG Pathways in the Optimal Set

We got eight KEGG pathway terms in the optimal set of features (see Supplementary S5), which are hsa00520 (amino sugar and nucleotide sugar metabolism) and has 00563 (glycosylphosphatidylinositol- (GPI-) anchor biosynthesis), hsa03015 (mRNA surveillance pathway), hsa03440 (homologous recombination), hsa03450 (nonhomologous end joining), hsa04110 (cell cycle), hsa04114 (oocyte meiosis), and hsa04330 (notch signaling pathway). Among them, amino sugar and nucleotide sugar metabolism (hsa00520) emphasize the sugar metabolism in eye cancer. Glycosylphosphatidylinositol- (GPI-) anchor biosynthesis (hsa00563) pathway is related with anchoring of proteins outside of membrane. The next three are all included in genetic information processing pathway. The mRNA surveillance pathway (hsa03015) involved in translation and the other two deal with replication and repair. Cell cycle (hsa04110) and oocyte meiosis (hsa04114) are related to cell growth and death, and notch signaling pathway (hsa04330) is involved in signal transduction.

The canonical pathway that links tumor suppressor gene Rb to human cancers details its interaction with the E2F transcription factors and cell-cycle progression [46]; recent studies have shown a significant role for RB-1 in the suppression of glycolytic and glutaminolytic metabolism [47, 48]. So the RB-E2F axis and the up- and down-stream genes should be very important in finding new potent antitumor target for Rb treatment.

## 4. Conclusion

We proposed a computational method to identify cancer related genes taking GO enrichment scores and KEGG enrichment scores as features. We applied this method to RB. An optimal feature set including 1061 GO terms and 8 KEGG pathways was revealed by our method, which has been shown to be closely related to RB. We believe this method is efficient and effective in prediction of novel cancer related genes and has universal applicability in the cancer research.

## Supplementary Material

Supplementary Material includes Supplementary S1: This file includes two sheets. The first one shows the differentially expressed genes in two previous RB studies and the 119 overlap RB genes. The second sheet shows all the positive samples and negative samples used in this study. The ration between positive and negative samples is 1:50. The corresponding Ensembl protein IDs were given. Supplementary S2: This file contains twenty sheets. For each dataset, there were two tables, of which one is MaxRel feature table ranked according to the relevance between the features and the class of the samples and the other one is the mRMR feature table ranked according to the redundancy and relevance to the features of the samples. Supplementary S3: The sensitivity (Sn), specificity (Sp), accuracy (Ac), Matthews's correlation coefficient (MCC) generated by each run of the IFS for each of the ten datasets. Supplementary S4: The final optimal 1069 features including 1061 GO terms and 8 KEGG pathways. Supplementary S5: IFS curves for the rest nine datasets. Supplementary S6: GO and KEGG enrichment result for 119 RB genes

## Figures and Tables

**Table 1:** The predicted results for ten datasets.

Dataset	Optimal feature number	Sn	Sp	Acc	MCC
1	156	0.5727	0.9291	0.8697	0.5174
2	141	0.6273	0.9218	0.8727	0.5452
3	337	0.7364	0.8691	0.8470	0.5347
4	140	0.6000	0.9327	0.8773	0.5471
5	126	0.5636	0.9436	0.8803	0.5434
6	489	0.6273	0.9255	0.8758	0.5527
7	78	0.5545	0.9527	0.8864	0.5588
8	222	0.6364	0.9345	0.8848	0.5795
9	319	0.6545	0.9218	0.8773	0.5663
10	235	0.5545	0.9491	0.8833	0.5495

Mean (standard deviation)		0.6127 (0.0567)	0.928 (0.0234)	0.8755 (0.0113)	0.5494 (0.017)

Sn: sensitivity; Sp: specificity; Acc: accuracy; MCC: Matthews's correlation coefficient.
