# Supplementary figures and images for: Prediction and Analysis of Retinoblastoma Related Genes through Gene Ontology and KEGG
Source: Biomed Res Int. 2013 Aug 13;2013:304029. doi: 10.1155/2013/304029 (PMC3755425; doi:10.1155/2013/304029)

**Dataset 2**

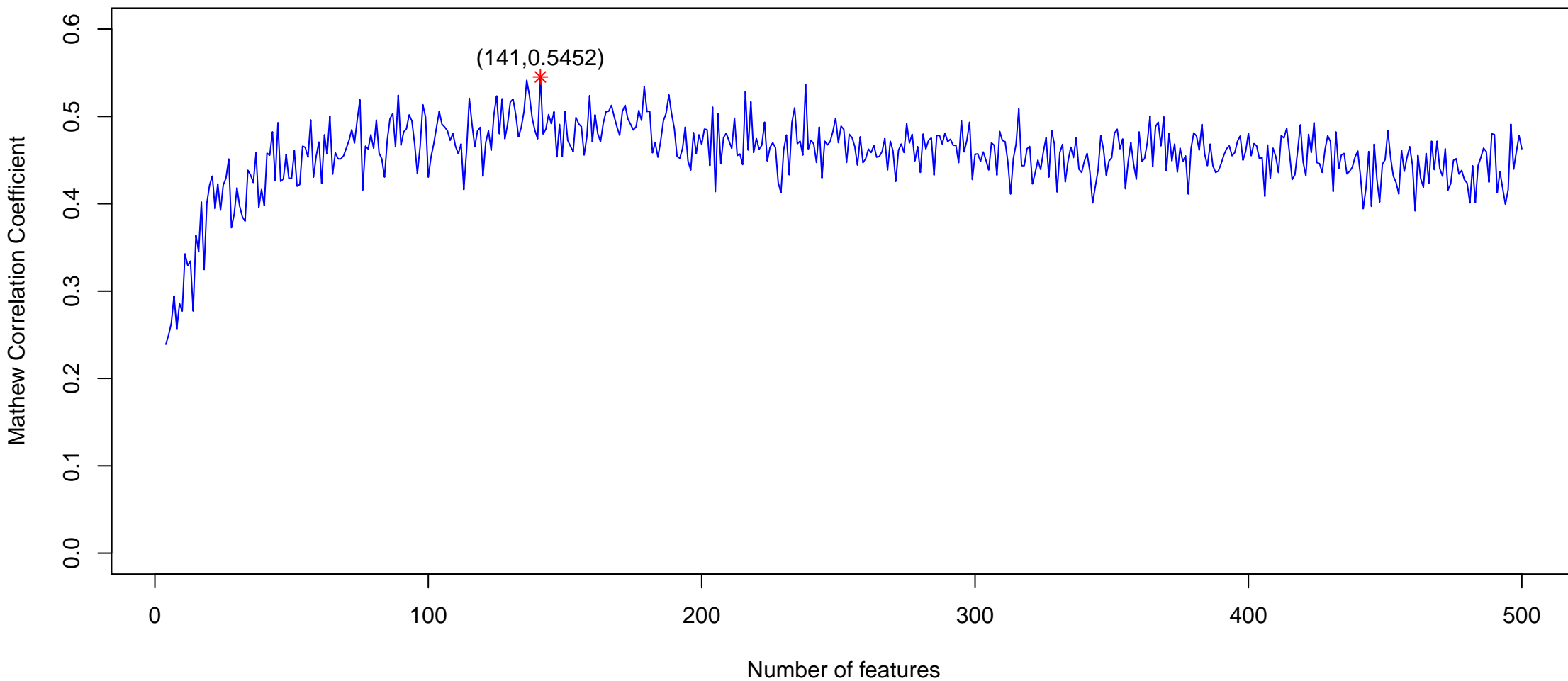

**Dataset 3**

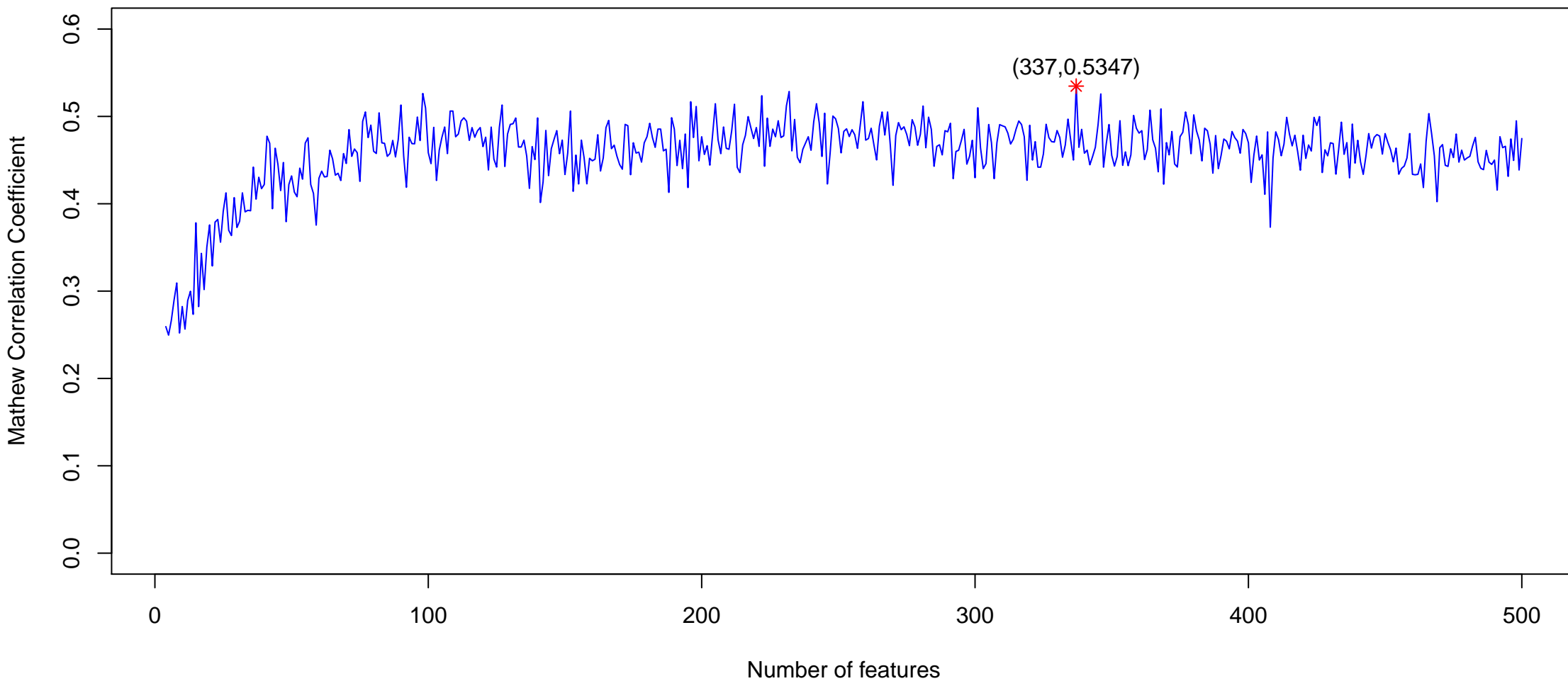

**Dataset 4**

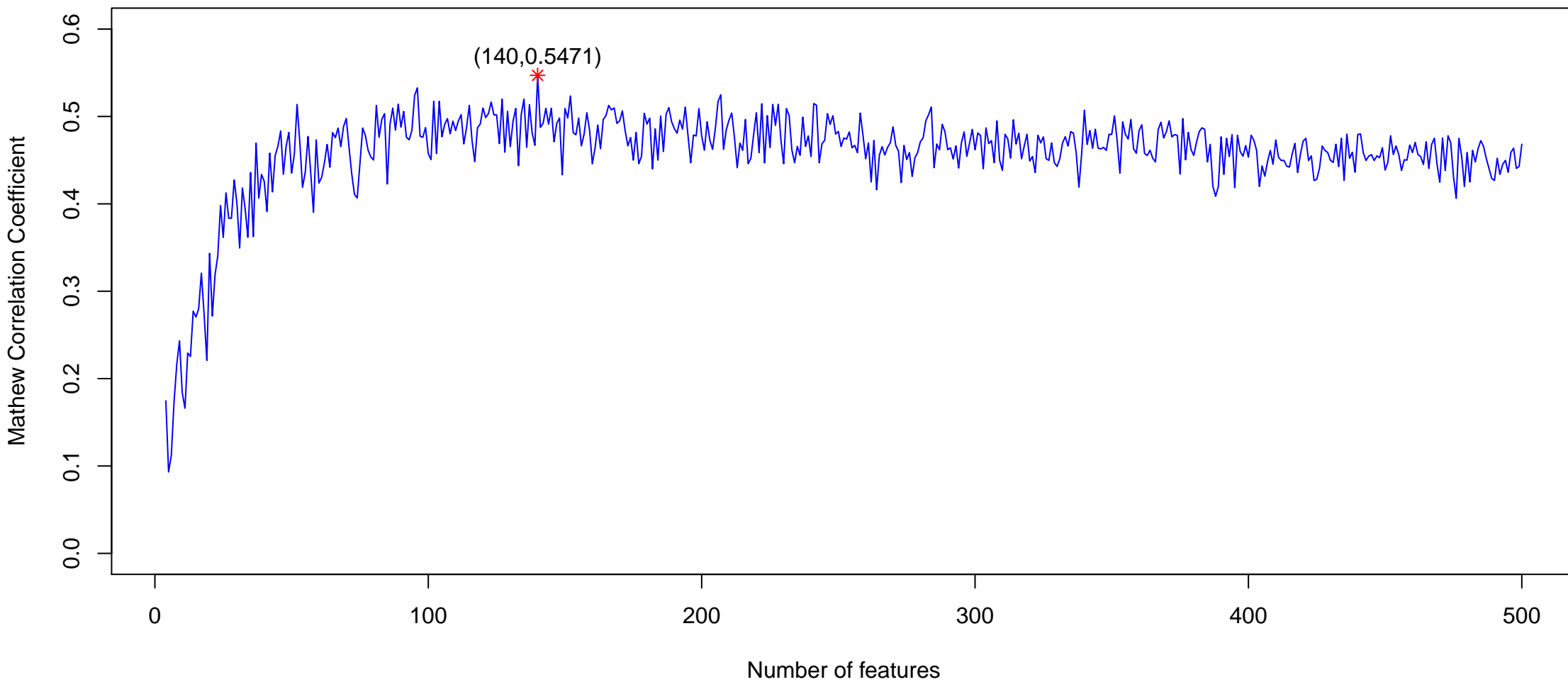

**Dataset 5**

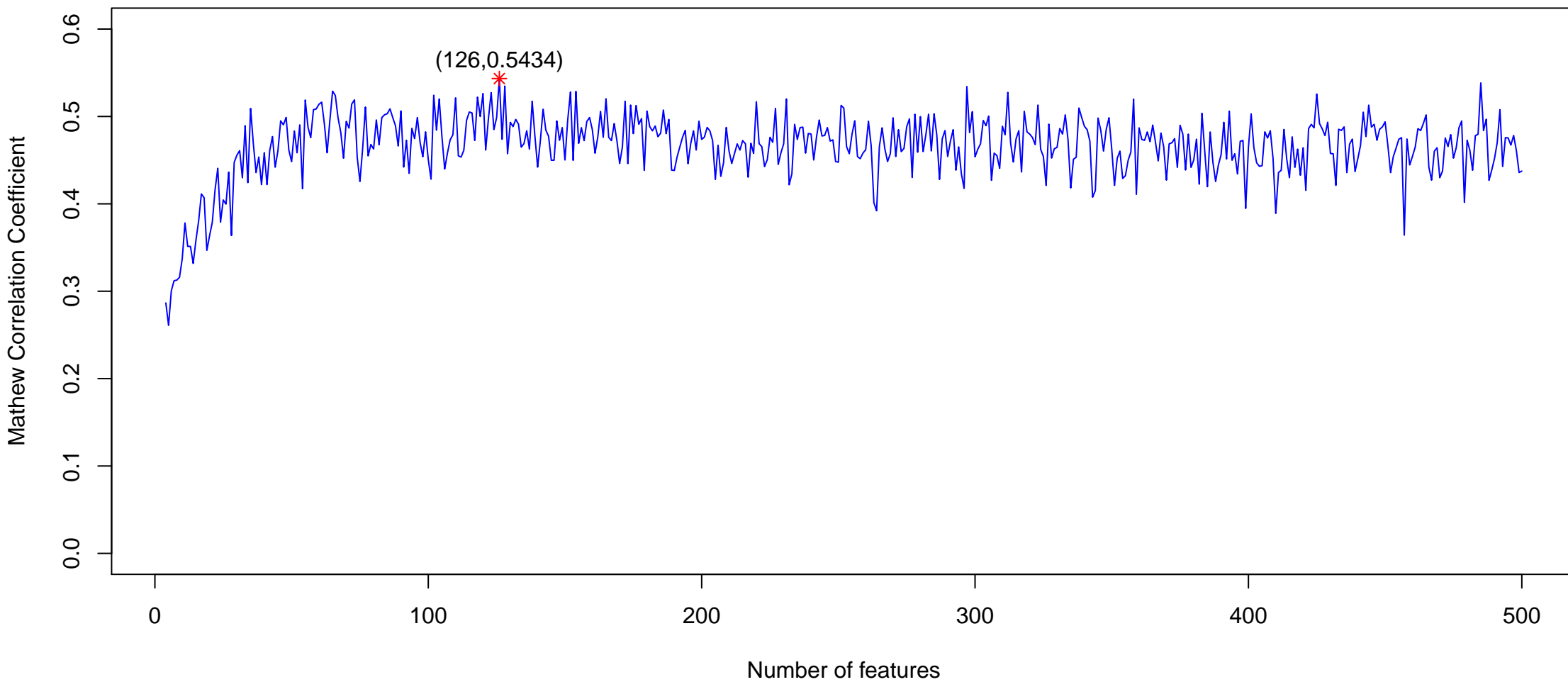

**Dataset 6**

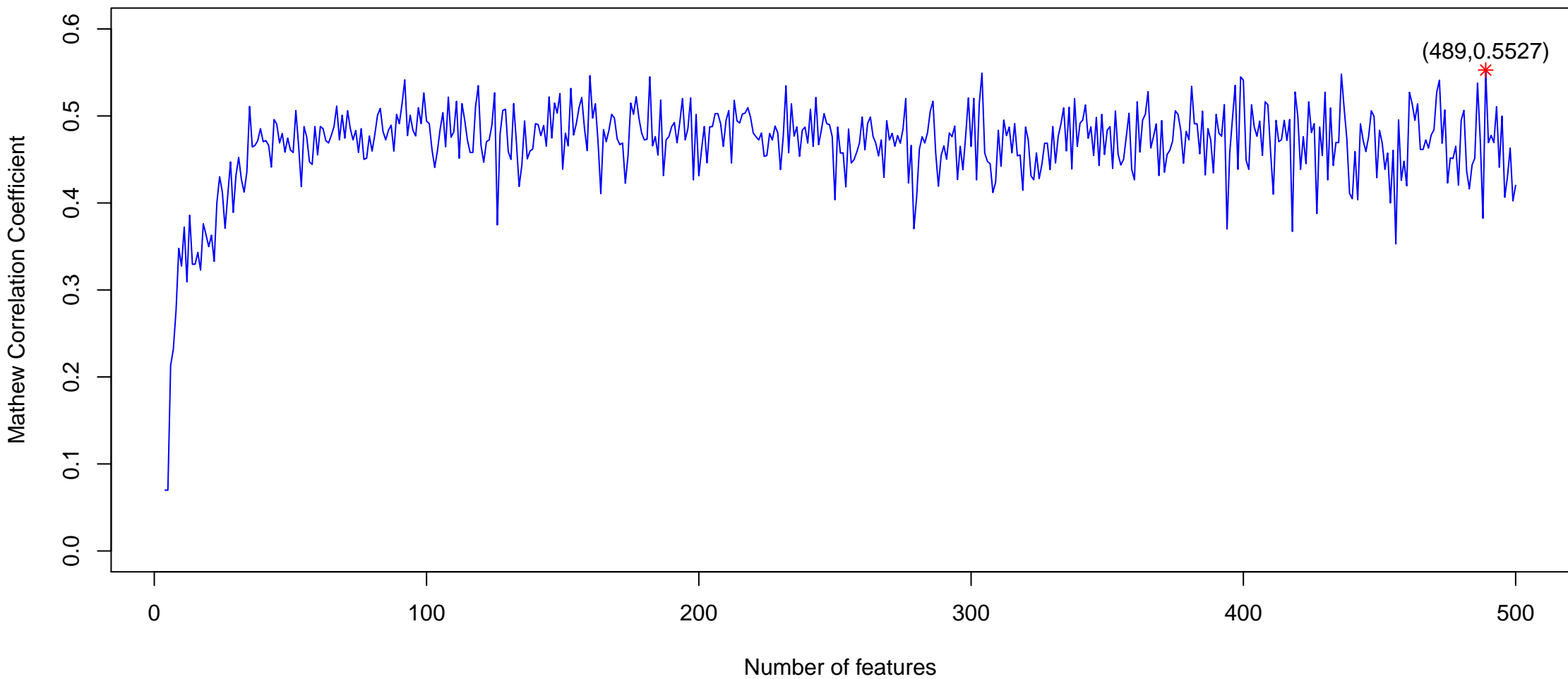

**Dataset 7**

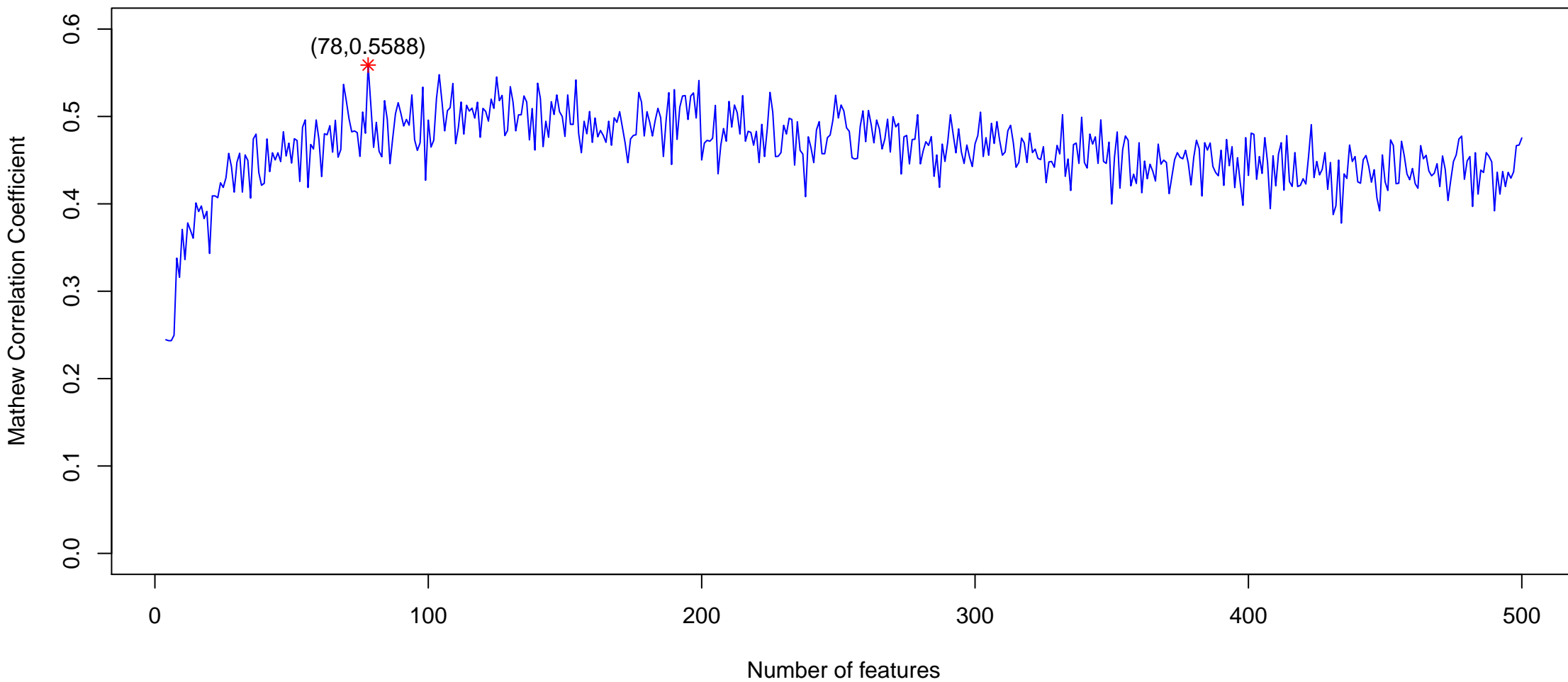

**Dataset 8**

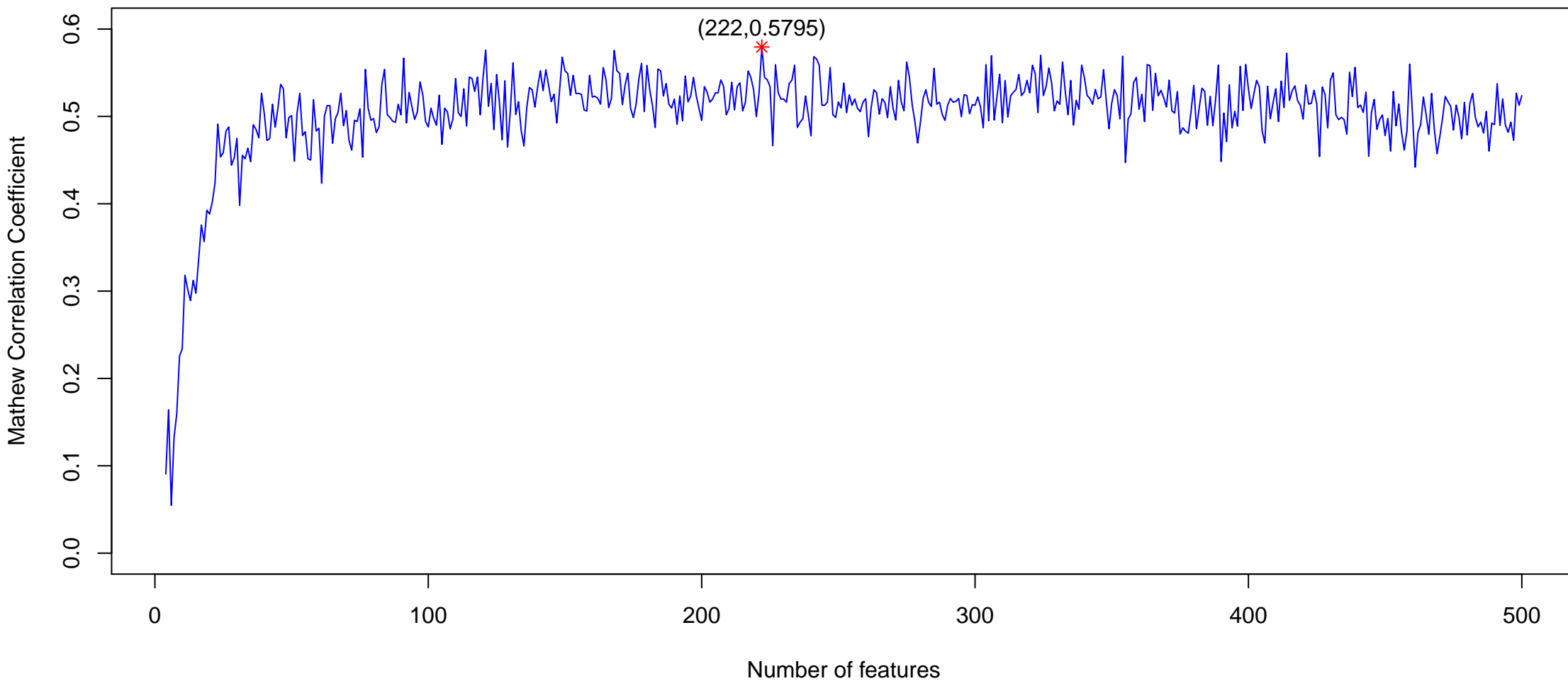

**Dataset 9**

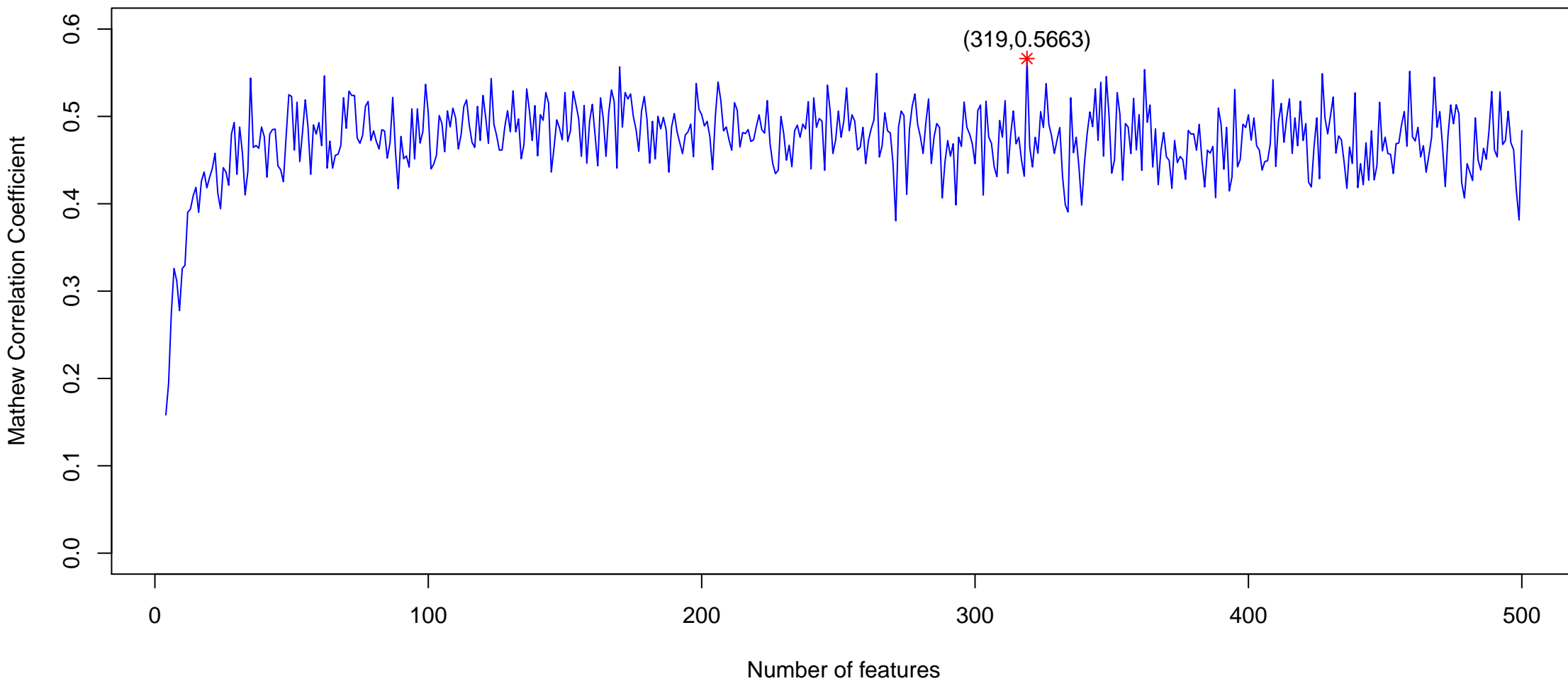

**Dataset 10**

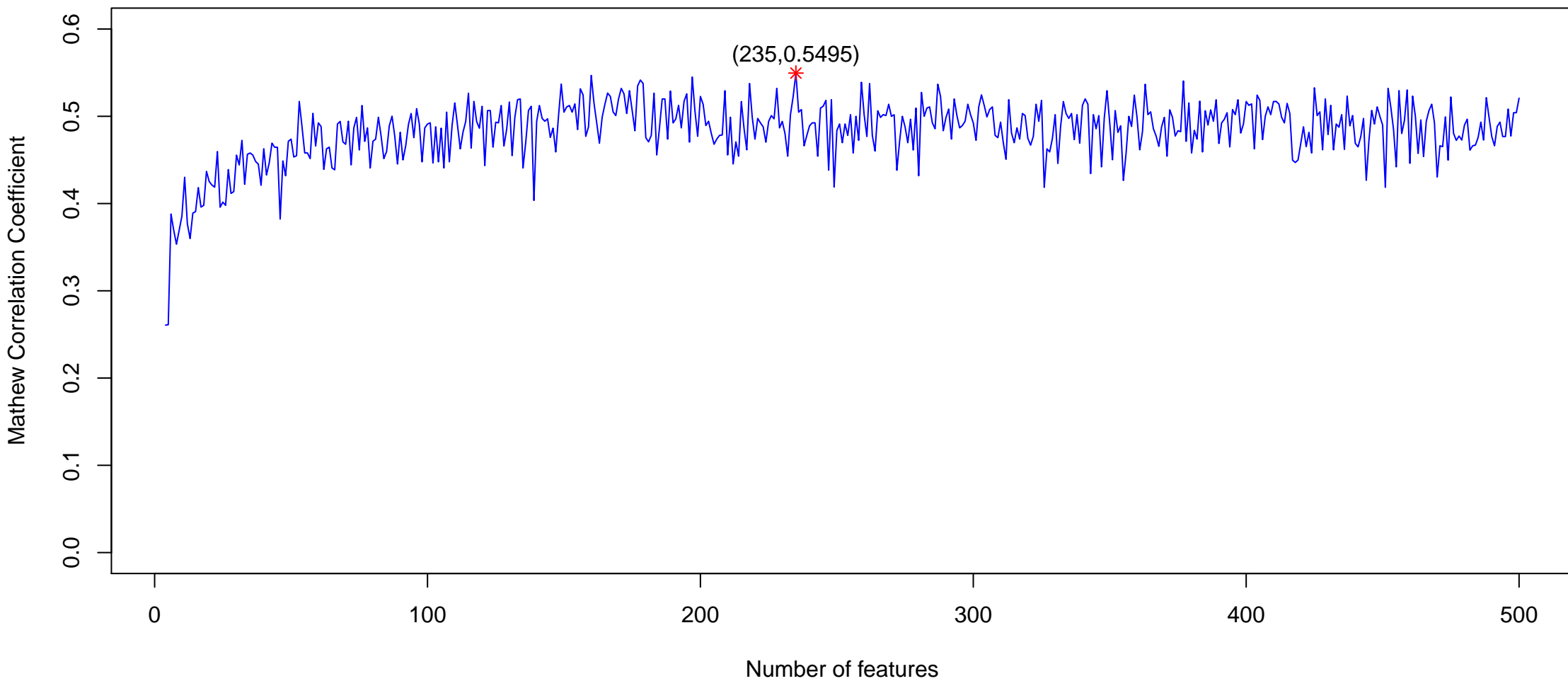

Supplement: Supplementary file 4 [file 304029.f4.pdf]
